# The Cytoprotective Role of Antioxidants in Mammalian Cells Under Rapidly Varying UV Conditions During Stratospheric Balloon Campaign

**DOI:** 10.3389/fphar.2019.00851

**Published:** 2019-08-02

**Authors:** Dawid Przystupski, Agata Górska, Paulina Rozborska, Weronika Bartosik, Olga Michel, Joanna Rossowska, Anna Szewczyk, Małgorzata Drąg-Zalesińska, Paulina Kasperkiewicz, Jędrzej Górski, Julita Kulbacka

**Affiliations:** ^1^Faculty of Medicine, Wroclaw Medical University, Wroclaw, Poland; ^2^Department of Biological Sciences, Institute of Experimental Biology, University of Wroclaw, Wroclaw, Poland; ^3^Faculty of Chemistry, Wroclaw University of Science and Technology, Wroclaw, Poland; ^4^Faculty of Biotechnology, University of Wroclaw, Wroclaw, Poland; ^5^Department of Medical Biochemistry, Wroclaw Medical University, Wroclaw, Poland; ^6^Ludwik Hirszfeld Institute of Immunology and Experimental Therapy, Polish Academy of Sciences, Wroclaw, Poland; ^7^Department of Molecular and Cellular Biology, Wroclaw Medical University, Wroclaw, Poland; ^8^Department of Animal Developmental Biology, Institute of Experimental Biology, University of Wroclaw, Wroclaw, Poland; ^9^Department of Human Morphology and Embryology, Wroclaw Medical University, Wroclaw, Poland; ^10^Department of Bioorganic Chemistry, Faculty of Chemistry, Wroclaw University of Science and Technology, Wroclaw, Poland; ^11^Faculty of Mechanical Engineering, Wroclaw University of Science and Technology, Wroclaw, Poland

**Keywords:** stratosphere, antioxidants, radiation, UV, oxidative stress, DNA damage, cell death

## Abstract

The current age of dynamic development of the space industry brings the mankind closer to routine manned space flights and space tourism. This progress leads to a demand for intensive astrobiological research aimed at improving strategies of the pharmacological protection of the human cells against extreme conditions. Although routine research in space remains out of our reach, it is worth noticing that the unique severe environment of the Earth’s stratosphere has been found to mimic subcosmic conditions, giving rise to the opportunity to use the stratospheric surface as a research model for the astrobiological studies. Our study included launching into the stratosphere a balloon containing mammalian normal and cancer cells treated with various compounds to examine whether these substances are capable of protecting the cells against stress caused by rapidly varying temperature, pressure, and radiation, especially UV. Owing to oxidative stress caused by irradiation and temperature shock, we used natural compounds which display antioxidant properties, namely, catechin isolated from green tea, honokiol derived from magnolia, curcumin from turmeric, and cinnamon extract. “After-flight” laboratory tests have shown the most active antioxidants as potential agents which can minimize harmful impact of extreme conditions on human cells.

## Introduction

**Figure 1:**
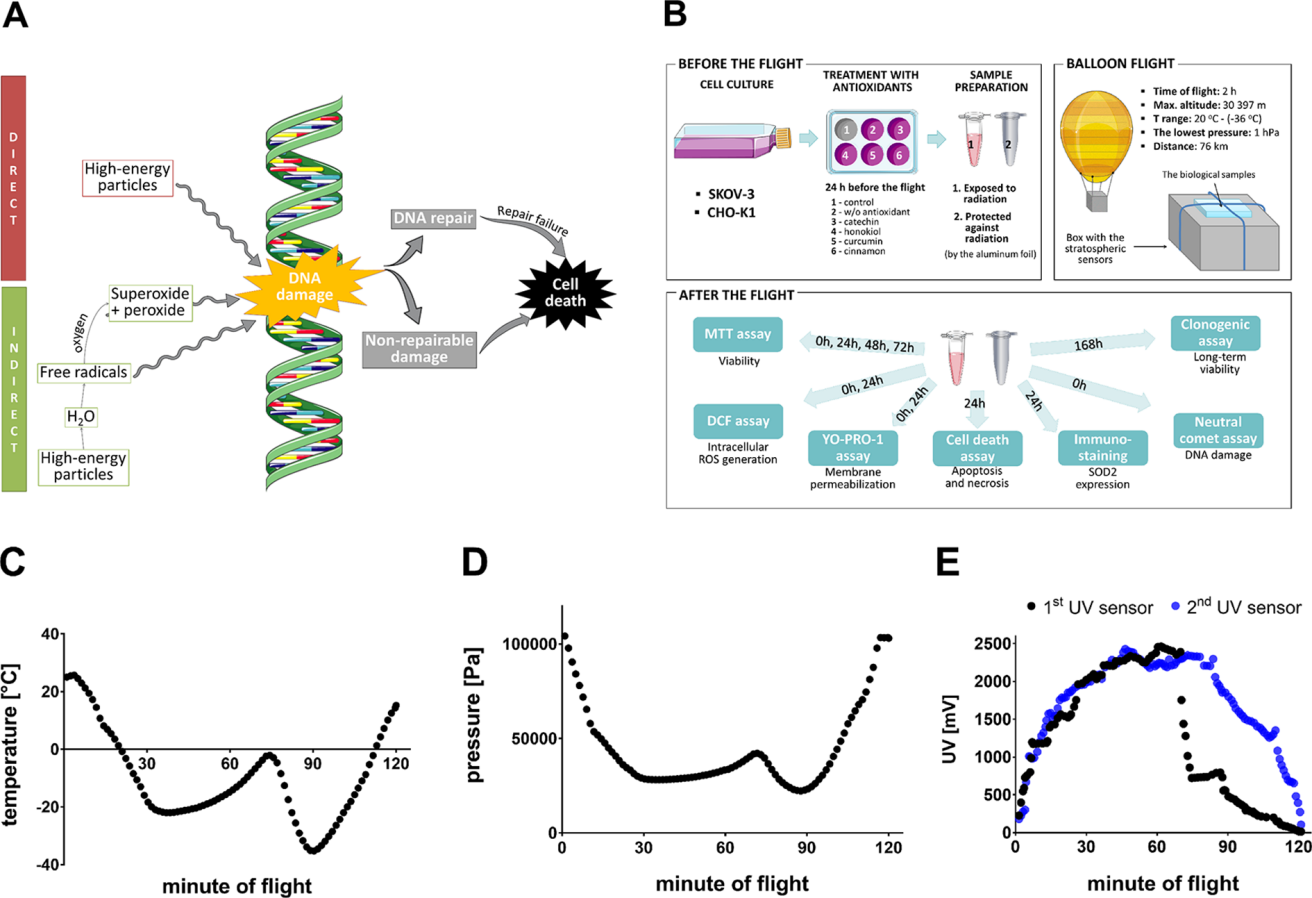
**(A)** Effect of the radiation on the DNA. High-energy particles could affect DNA damage either by direct pathway or indirect pathway. Free radicals and by-products of the process of water radiolysis contribute to an increase in the oxidative stress in the cell affecting the DNA. Survival of the cell depends on the extent of the DNA damage (Anand et al., 1997; Cohen-Jonathan et al., 1999; Desouky et al., 2015). **(B)** The schematic representation of the procedure of experiment and balloon flight. **(C)** The temperature fluctuations (°C) during the balloon flight outside the payload; the sensor was located at the top wall of gondola. **(D)** The pressure fluctuations (Pa) during the balloon flight; the sensor was located at the top wall of gondola. **(E)** UV radiation fluctuations (mV) during the balloon flight. The first UV sensor was located at the top wall of the gondola, the second UV sensor measured UV radiation at the side wall. This figure was prepared using Servier Medical Art, available from www.servier.com/Powerpoint-image-bank.

The world’s first hydrogen balloon was created over 200 years ago in Paris, which started the era of scientific ballooning (Petrescu and Petrescu, 2012). Dynamic development of this technique enabled the advanced research in atmospheric science, aerobiology, and meteorology (Jones, 2014) to flourish, remaining a promising tool in astrobiology and space biology. Stratospheric balloon flights allow to mimic Mars-like conditions, which is why they have been used to analyse the effect of the exposure of the polyextremophilic species to the harsh environment of upper atmosphere (Smith, 2013; Smith et al., 2014; Caro et al., 2019). In the stratosphere, the temperature reaches −40°C, atmospheric pressure is at 1 kPa, which is roughly equivalent to the surface pressure on Mars (0.5–1 kPa), the relative humidity of air is lower than 1%, solar UV irradiance is ∼100 W/m^2^, and cosmic radiation is at the level of 0.1 mGy/day (Murgatroyd, 1970; Reitz, 1993; Smith and Sowa, 2017). Furthermore, the UV irradiation in the stratosphere has been proven to resemble the model UV radiation levels on the surface of Mars, especially in the wavelength range between 250 and 400 nm (Schuerger et al., 2003). In recent years, specialized laboratory chambers have been designed to mimic Martian conditions; however, they have failed to accurately imitate Martian conditions because of their incapability to represent the dynamic nature of sunlight and create the full range of biology-relevant factors present on Mars (low temperature, pressure and humidity, radiation, oxidation, etc.) (Diaz and Schulze-Makuch, 2006; Moores et al., 2007; Fendrihan et al., 2009; Smith et al., 2009; Gómez et al., 2010; Peeters et al., 2010; Johnson et al., 2011;Kerney and Schuerger, 2011). The unique stratospheric environment exhibits natural combination of these severe conditions, which is why it may be utilized to investigate the effect of subcosmic and Martian conditions on biological processes (Wong et al., 2017). Moreover, stratospheric flights could potentially provide us with valuable information about the stress response in living organisms after exposure to rapidly varying different severe environmental factors at the same time. It allows us to examine whether some compounds are able to support the viability of living organisms and cells in such extreme environment. The exposure of varied biological samples to the stratospheric conditions opens up the possibility to observe changes in the functioning of the cell, e.g., decreased viability, dysfunction of cellular organelles and their localization, cell cycle arrest, changes of gene expression, and DNA damage (Moan and Peak, 1989; Cadet et al., 2005). In addition, ionizing and nonionizing radiation in the stratosphere (Dachev, 2013) affects the cells both directly and indirectly [water radiolysis, therefore exacerbating the oxidative stress (Desouky et al., 2015)], causing effects such as mitochondrial impairment (Anand et al., 1997), DNA damage, protein and lipid peroxidation correlated with disruption of the cell membrane (Cohen-Jonathan et al., 1999) that altogether may lead to cell death (Cohen-Jonathan et al., 1999; Desouky et al., 2015) (**Figure 1A**). Furthermore, stratospheric flights provide unique cyclic changes of linked environmental factors, including radiation, overload, pressure, temperature, wind (high velocities, rapidly varying wind direction), and vibrations, which are impossible to be simulated altogether in laboratory. The wide use of balloons provides numerous advantages including lower overall project costs, recoverable and massive payloads (up to 3,600 kg), and more rapid and flexible flight (Smith and Sowa, 2017).

It is known that natural substances derived from plants exhibit positive impact on living cells, including their protective role against diverse factors generating oxidative stress. Green tea polyphenols, such as epigallocatechin gallate, epicatechin gallate, epigallocatechin, epicatechin, and catechin (Narumi et al., 2014), are one of the strongest antioxidants. The long-term consumption of green tea extracts not only increases the activity of oxidative stress enzymes (Song et al., 2014) (e.g., superoxide dismutase) and inhibits lipo- and cyclooxygenase, xanthine oxidase, activator protein 1, and NF-κB transcription factors activity (Hosseinimehr et al., 2013) but also protects cells against radiation (Vergne et al., 2018). Previous meta-analyses suggest a protective role of green tea against numerous cancer types (Kurahashi et al., 2007; Li et al., 2008; Shrubsole et al., 2009; Kang et al., 2010; Yang et al., 2011; Ni et al., 2017). Honokiol exhibits similar properties (Mihara et al., 2017; Banik et al., 2019). This magnolia-derived compound inhibits UVB-induced immunosuppression and induces apoptosis in malignant cells (Prasad et al., 2017). Chilampalli et al. (2010) showed that pretreatment with honokiol is efficient in preventing skin carcinogenesis induced by UV (Chilampalli et al., 2010). Furthermore, honokiol inhibits photocarcinogenesis by targeting UVB-induced inflammatory mediators and cell cycle regulator (Vaid et al., 2010; Chilampalli et al., 2011). Turmeric compounds are another group of free radicals’ scavengers. Curcumin displays anti-inflammatory (Ni et al., 2015), antibacterial (Koosirirat et al., 2010), antiviral (Moghadamtousi et al., 2014), anticancer (Sinha et al., 2012), proapoptotic (Wang et al., 2011), neuroprotective (Khanna et al., 2009; Darvesh et al., 2012a), and hepatoprotective (Darvesh et al., 2012b) activity, promotes autophagy (Zhang et al., 2016), and affects the cytochrome P-450 enzyme pathway (Hsieh et al., 2014) and phase II enzymes (Das and Vinayak, 2015). Curcumin is able to neutralize different forms of free radicals, such as reactive oxygen and nitrogen species (Menon and Sudheer, 2007), and modulate the activity of GSH, catalase, and SOD enzymes, which reduce oxidative stress (Lin et al., 2007; Marchiani et al., 2014). It can inhibit reactive oxygen species (ROS)-generating enzymes such as lipoxygenase/cyclooxygenase and xanthine hydrogenase/oxidase (Lin et al., 2007; Choi et al., 2017; Hewlings and Kalman, 2017). Furthermore, curcumin indicates photosensitizing properties—it has an ability to absorb radiation in a particular wavelength, sensitizing the tissue to light and promoting cell death. Therefore, it can act as a photosensitizer (Verwanger et al., 2011). Thus, due to widely reported photophysical and photochemical properties (Priyadarsini, 2009; Banerjee et al., 2012; Sarkar and Hussain, 2016; Machado et al., 2019) as well as cytotoxicity restricted to cancer cells with no harm to normal cells (Aggarwal et al., 2003; Sharma et al., 2004; Hsu and Cheng, 2007; Dhillon et al., 2008), curcumin has already been applied in cancer therapy as photochemotherapeutic agent (Duse et al., 2018; Khorsandi et al., 2019; Roos et al., 2019). In conclusion, unique characteristics of this compound make it an exceptionally promising protective agent in extreme conditions. Different forms of free radicals can be scavenged by turmeric compounds. Similar properties are displayed by organic compounds found in cinnamon extract. In addition to the antioxidant activity, cinnamon presents radioprotective potential. Azab et al. (2011) have revealed that administration of cinnamon extract in irradiated rats decreased ROS production by inhibition of NF-κB activation as well as its polyphenols contents (Azab et al., 2011).

Regarding the antioxidant-mediated handling with extreme conditions, it is worth to note the impact of the compounds such as cinnamon (Azab et al., 2011), curcumin (Hung et al., 2008; Barry et al., 2009; Tsukamoto et al., 2014; Ben-Zichri et al., 2019), plant flavonoids (Oteiza et al., 2005; Ajdžanović et al., 2013; Tarahovsky et al., 2014), or polyphenols (Cyboran et al., 2012) on the biophysical properties of cell membrane, which could play a protective role in a rapidly changing environment.

Thus, the primary objective of this study was to evaluate the effect of stratospheric environment, namely, rapidly varying UV, on two living cell lines: human ovarian cancer cells (SKOV-3) and Chinese hamster ovary cells (CHO-K1), which were used as a noncancer research model (**Figure 1B**). The cells were treated with various antioxidants (catechin isolated from green tea, honokiol derived from magnolia, curcumin from turmeric, and cinnamon extract) to examine whether these substances are capable of protecting the cells from stress caused by irradiation and temperature shock during stratospheric balloon flight. “After-flight” laboratory tests displayed the most active antioxidants as potential agents which can minimize harmful impact of extreme conditions on mammalian cells. Apart from numerous differences in mechanisms of action of each particular antioxidative agent, final evaluation of their protective properties is reflected in the mitigation of the genotoxicity caused by combination of varying unfavorable factors such as temperature and UV radiation.

## Materials and Methods

### Balloon Flight and Atmospheric Conditions Analysis

The balloon was filled with helium, and its position was tracked by APRS-supported services: www.habhub.com and www.aprs.fi. The telemetry was emitted through RTTY 70 cm/APRS 2 m signal, supported with a GPS/GSM tracker. On-board computer (ATMEGA328P) was equipped with sensors located at the top wall [for UV radiation (ML8511), pressure (BMP085), and temperature (DHT22) measurements], additional UV sensors at the side wall of the gondola, and accelerometer (MPU6050) inside the box. To compare stratospheric and Earth UV irradiation, the UV ML8511 sensor was used—designed especially for surface measurement of UV spectra [UVA (280–400 nm) and UVB (260–280 nm)]. As it is mentioned in the specifications, this sensor has a nominal operation range of −20–70°C, suggesting that the monitoring of UV would only be valid up to roughly the minute 30 of the flight. This means that some of the random variations depicted in **Figure 1E** may be an artifact of the electronics, which is not operating adequately at such low temperatures. The electronic devices oftentimes produce too much noise when going below certain temperature thresholds and furthermore being exposed to moisture freezing. However, ground analyses displayed that after recalibration, the sensors may be used in temperatures below −20°C, which caused insignificant disturbances of measurements.

The biological samples were launched to the stratosphere on the 30th of April 2018, from Wrocław, Poland (51°06′23.6″ N 17°03′32″ E) at 11:30 am. The balloon reached the stratosphere at maximal altitude of 30,298 m. The mission lasted ∼2 h: 90 min of ascent and 25 min of descent; ended at 1:25 pm. The biological samples were collected immediately after landing in Sulisław, Poland (52°23′49.9″ N 18°45′52.8″ E) and transported directly to the laboratory. During the first stage of ascending phase, recorded ambient temperature dropped to −22°C. Subsequently, when the balloon reached the ozonosphere, the ambient temperature increased to −2°C. At the highest altitude, the temperature reached the lowest level of −35°C (**Figure 1C**), and the lowest pressure (1,252 Pa) was measured (**Figure 1D**). Data provided by two UV sensors showed that the top and side walls of the gondola were similarly exposed to the UV radiation during the balloon ascent. However, during the balloon descent, there was a difference observed between the measured UV radiation on both sides of the gondola, caused by continuous rotations of the gondola in the last stage of the flight. (**Figure 1E**). Voltage level of 1,170 mV correlates with the highest score (11) in the UV Index exposition scale, which shows extreme exposure to the UV radiation, causing immediate damage of unprotected human skin and eyes (Fioletov et al., 2010). On the right side of the chart, there is a clearly visible period measured by the first UV sensor when the parachute was opened; as a result, the gondola was stabilized. In the upper parts of the atmosphere, the UV dose was more than twofold the dose correlating with the maximum dose in the UV Index scale (reaching nearly 2,463 mV).

### Experimental Protocol

First, the adherent cells were seeded at equal density (3 × 10^4^ cells/cm^2^) on six-well plates. After 24-h incubation with various antioxidants, the cells were detached, suspended in freezing medium Bambanker^™^ (Nippon Genetics, Cat. no. BB01) (1,5 × 10^6^ cells/300 μl), and placed in microtubes 30 min before the balloon flight. Then, the samples were transported on ice to the starting point and placed in a radiation transmitting gondola, located on the environmental measurement unit with accelerometer and temperature, pressure, and UV sensors. One-half of the samples was covered with aluminum foil to protect the cells against irradiation, mostly UV (the samples described in the study as “protected against radiation”); another half was sent into the stratosphere without the protective layer (described as “not protected against radiation”). As a result, we were able to evaluate the effect of radiation on examined cells in the presence of various antioxidants. As a control, we used the appropriate number of cells not treated with antioxidants and not sent into the stratosphere, which were incubated at 37°C in a humidified incubator with 5% CO_2_ during the flight. Directly after landing, the biological samples were transported on ice to the specialized laboratory, where after-flight tests were performed. The cells were seeded on 96-well plates (3 × 10^4^ cells/cm^2^) and incubated in an appropriate drug solution for 24 h to perform membrane permeabilization assay and intracellular ROS generation assay, as well as for 24, 48, and 72 h to evaluate mitochondrial activity in MTT assay. Modified versions of these assays (adding reagents into the cells’ suspension, appropriate incubation, centrifugation, and seeding on 96-well plates—3 × 10^5^ cells/cm^5^) were used to analyze the suspended (not adherent to multiwell plates) cells directly after the balloon landing (0 h). In addition, the cells were plated on six-well plates and incubated in the appropriate drug solution for 24 h to perform cell death assay (cell density seeding 3 × 10^4^ cells/cm^2^) and for 7 days to carry out clonogenic assay (cell density seeding 15 cells/cm^2^). To analyze the expression of manganese-dependent superoxide dismutase (SOD2), the cells were seeded on 10-well diagnostic microscopic slides (3 × 10^4^ cells/cm^2^) and fixed after 24 h. Immunocytochemical staining was carried out in the two following days. Furthermore, neutral comet assay was performed to analyze DNA damages associated with the balloon flight. The scheme of the completed experiment is presented on **Figure 1B**.

### Cell Culture

Human ovarian cancer cells (SKOV-3; described as “cancer cells”) and noncancer Chinese hamster ovary cells (CHO-K1; described as “normal cells”) were obtained from the American Type Culture Collection (ATCC, London, UK). Cells were cultured as a monolayer in Dulbecco’s modified Eagle medium (DMEM, Sigma-Aldrich, USA; for SKOV-3 cells) and Ham’s F-10 Nutrient Mix (F-10, Sigma-Aldrich, USA; for CHO-K1 cells) containing 2 mM L-glutamine, 10% fetal bovine serum (FBS, Sigma-Aldrich), 20 U penicillin, and 20 µg streptomycin/ml (Sigma-Aldrich) at 37°C in a humidified incubator with 5% CO_2_. For the experiments, the cells were washed with Dulbecco’s phosphate-buffered saline (PBS, Bioshop, UK) and detached from the flask’s surface using 0.25% trypsin with 0.02% EDTA (Sigma-Aldrich, Poland).

### Chemical Substances

For the experiments, we used four compounds with antioxidative potential: (±)-catechin hydrate (Sigma, Cat. no. C1788), curcumin (Sigma-Aldrich, Cat. no. C1386), honokiol (Sigma-Aldrich, Cat. no. H4914), and cinnamon oil (Avicenna-Oil, Cat. no. 1011/5). Each drug solution was prepared directly before the experiment. Catechin was first dissolved in 96% ethanol to give a stock solution of 5 mM concentration and afterwards diluted to 10 μM concentration with culture medium. Honokiol and curcumin were dissolved in dimethyl sulfoxide (DMSO, Sigma-Aldrich, Cat. no. D8418) to the 5 mM concentration and subsequently diluted to 10 μM (curcumin) and 2 μM (honokiol) concentrations with growth medium. Cinnamon was prepared by dilution in the PBS buffer to the 1,000 μg/l concentration, then diluted again to 2 μg/l concentration with culture medium. The final concentration of the solvent was <2% and did not alter statistically the cell viability.

### MTT Assay

To analyze cell viability, the mitochondrial activity was measured using the standard MTT [3-(4,5-dimethylthiazol-2-yl)-2,5-diphenyltetrazolium bromide] assay (MTT, Sigma-Aldrich). The cells were incubated on a 96-well plate (Perkin Merkel) in the concentration of 10,000 cells/well for 3 h (37°C) in 0.5 mg/ml MTT solution in PBS buffer (PBS, Bioshop, UK) 100 μl/well. The absorbance was measured in 0 h (directly after the balloon flight), 24, 48, and 72 h incubation after balloon landing at 570 nm (Multiplate reader EnSpire, Perkin Elmer). Acidified isopropanol (100 μl/well, 0.04 M HCl in absolute isopropanol per well) was used to dissolve the formed formazan crystals.

### Cell Death Assay

After landing, the cells were plated on six-well plates (3 × 10^4^ cells/cm^2^) and incubated for 24 h in the appropriate drug solution. Following incubation, the culture medium was collected, and the cells were washed with PBS buffer and detached from the surface using trypsin-EDTA solution. The collected growth medium and cell suspension were centrifuged (6,720 × *g*) for 10 min at 4°C. Subsequently, they were stained with Annexin V-APC Apoptosis Kit with PI (BioLegend, Cat. no. 640932) and analyzed with FACS Calibur flow cytometer (Becton Dickinson) to indicate the percentage of early and late apoptotic and necrotic cells.

### Membrane Permeabilization Assay

Fluorescent dye YO-PRO-1 (Invitrogen, Cat. no. Y3603) was used to evaluate the plasma membrane permeabilization caused by low temperature and radiation cell damage during the balloon flight. The cells were seeded on black 96-well plates (3 × 10^4^ cells/cm^2^) and stained in 1 μM YO-PRO-1 diluted in growth medium for 10 min. After 10 min of incubation and washing with PBS, the mean intracellular fluorescence of YO-PRO-1 was measured with excitation wavelength of 491 nm and the emission wavelength of 509 nm using a plate reader (Multiplate Reader EnSpire, Perkin Elmer). Membrane permeabilization assay was evaluated directly after balloon flight (0 h) and 24 h after landing (24 h).

### Intracellular ROS Generation Assay

The level of the ROS in cells was determined using the DCF (2,7-dichlorofluorescein) assay (Life Technologies, Poland). For experiments, the stock solution of carboxy-H_2_DCFDA (50 µg/ml in sterile DMSO; Sigma, Poland) was established at the RT in the dark and then diluted in a cell culture medium without FBS. The cells were seeded on black 96-well plates (3 × 10^4^ cells/cm^2^). After washing with PBS, the reagent was added to the cell culture to a final concentration of 5 µM, and cells were incubated at 37°C in darkness for 30 min. After the incubation, the mean fluorescence of DCF in wells was measured every 30 min for 90 min total using a plate reader (Multiplate Reader EnSpire, Perkin Elmer) with excitation wavelength of 495 nm and emission wavelength of 530 nm. Intracellular ROS generation assay was evaluated directly after balloon flight (0 h) and 24 h after landing (24 h).

### Immunocytochemical Staining

Immunocytochemical staining was performed using the ABC method to investigate the effect of stratospheric conditions and antioxidative drugs on the expression of manganese-dependent superoxide dismutase (SOD2) in SKOV-3 and CHO-K1 cells. After landing, the cells were seeded on 10-well diagnostic microscopic slides (Thermo Fisher Scientific) and incubated for 24 h in the appropriate drug solution (1 × 10^4^ cells/cm^2^). After incubation, the cells were fixed and dehydrated using 4% paraformaldehyde (PFA, Sigma-Aldrich) for 10 min. Then, the cells were stained using the EXPOSE Mouse and Rabbit Specific HRP/DAB Detection IHC kit (Abcam, United States; Cat. no. ab80436). The enzyme expression was visualized with the mouse monoclonal antibody anti-SOD2 (Santa Cruz, USA, Cat. no. sc-362300) diluted 1:200 with the PBS buffer. After overnight incubation with the primary antibody, the cells were incubated with the secondary antibody conjugated with horseradish peroxidase (HRP). Next, samples were incubated with a diaminobenzidine–H_2_O_2_ mixture to visualize the HRP label. Between the steps, samples were rinsed using 1% Triton X-100 in PBS. The cells were stained with hematoxylin for 3 min to visualize the nuclei. The immunocytochemical reaction was evaluated with a double-blind method using an upright microscope (Olympus BX51, Japan). Then, the intensity of immunocytochemical reaction was evaluated using an image analysis software (ImageJ 1.43j, National Institutes of Health, Bethesda, Maryland, USA) and method described by Nguyen et al. (2013), allowing to measure mean intensity value of DAB in each cell (Nguyen et al., 2013), which corresponds to mean SOD2 antibody immunoreactivity and SOD2 expression in cells. The results are presented as a percentage ratio of SOD2 antibody immunoreactivity measured in control, nontreated cells.

### Clonogenic Assay

Clonogenic assay is a technique allowing for the assessment of cell survival and proliferation following the exposition to the tested compounds. After balloon landing, the cells were plated in appropriate dilutions (15 cells/cm^2^) into six-well plates at appropriate drug solution. Multiwell plates were placed in an incubator and left there for 7 days until large colonies (>1 mm) were formed (50 cells or more). After incubation, the growth medium was removed, and the cells were washed with PBS. Fixation and staining of clones were done with a mixture of 0.5% crystal violet in 4% paraformaldehyde (PFA, Sigma-Aldrich, USA) for 10 min. Then, the plates were rinsed with water and left to dry at room temperature. Counting of clones was performed the following day using the ImageJ software.

### Neutral Comet Assay

For detection of DNA damages associated with the exposure to extreme environment during the balloon flight, the neutral comet assay method described by Collins (Collins, 2002) was used. Directly after the balloon flight, CHO-K1 and SKOV-3 cells were suspended in freezing medium Bambanker^™^ (Nippon Genetics, Cat. no. BB01) (1 × 10^5^ cells/50 μl) and frozen for further analysis. After defrosting and centrifugation with PBS (10 min at 4°C, 6,720 × *g*), the cells at the concentration 1 × 10^5^/ml were mixed with low temperature melting agarose (Sigma) at the ratio 1:10 (v/v) and spread on a slide (1 × 10^2^ cells/cm^2^). Slides were submerged in precooled lytic solution (2.5 M NaCl, 100 mM EDTA, pH 10, 10 mM Tris base, and 1% Triton X-100) at 4°C for 60 min. After lysis and rinsing, slides were equilibrated in TBE solution (40 mM Tris/boric acid, 2 mM EDTA, pH 8.3), electrophoresed at 1.2 V/cm for 15 min; then, silver staining was performed (Nadin et al., 2001). For scoring the comet patterns, 200–300 nuclei from each slide were assessed. In addition, the samples were stained using fluorescent dye Green Dead Cell Stain (Thermo Fisher Scientific, Cat. no. S34860). The images were acquired on a fluorescence microscope (Olympus Nikon). For each measurement, at least 50 cells per sample were counted using the ImageJ software; the CometScore 2.0 software was used to analyze the comets. The tail DNA percentage was taken as a quantified index of DNA damage.

### Confocal Microscopy

Confocal laser scanning microscopy (CLMS) was used for the visualization of cell membrane damage and morphology. After the balloon flight, the cells were grown on coverslips for 24 h (1 × 10^4^ cells/cm^2^). Subsequently, the cells were washed three times with PBS and quickly submerged in staining solution CellMask^™^ Deep Red Plasma Membrane Stains (at a concentration of 0.5 μg/ml dissolved in growth medium; Molecular Probes, Cat. no. C10046, Ex./Em. 649/666 nm) for 10 min at 37°C. Next, the staining solution was removed, and the coverslips were rinsed with PBS three times. After washing out with PBS, nuclear DNA was stained with DAPI (4,6-diamidino-2-phenylindole; 0.2 μg/ml, Ex./Em. 358/461 nm). At the end, the cells were mounted in fluorescence mounting medium (DAKO). For imaging, Olympus FluoView FV1000 confocal laser scanning microscope (Olympus) was used. The images were recorded by employing a Plan-Apochromat 60× oil-immersion objective.

### Statistics

Statistical significance was determined by one-way ANOVA with Tukey *post hoc* test within groups following normal distribution or Kruskal–Wallis with Dunn’s *post hoc* test in case of not normally distributed values. Differences between treated samples and control cells with *p* ≤ 0.05 were assumed to be statistically significant. The results were analyzed with the Microsoft Office Excel 2017 and GraphPad Prism 7.0 software.

## Results

### Mitochondrial Activity

**Figure 2:**
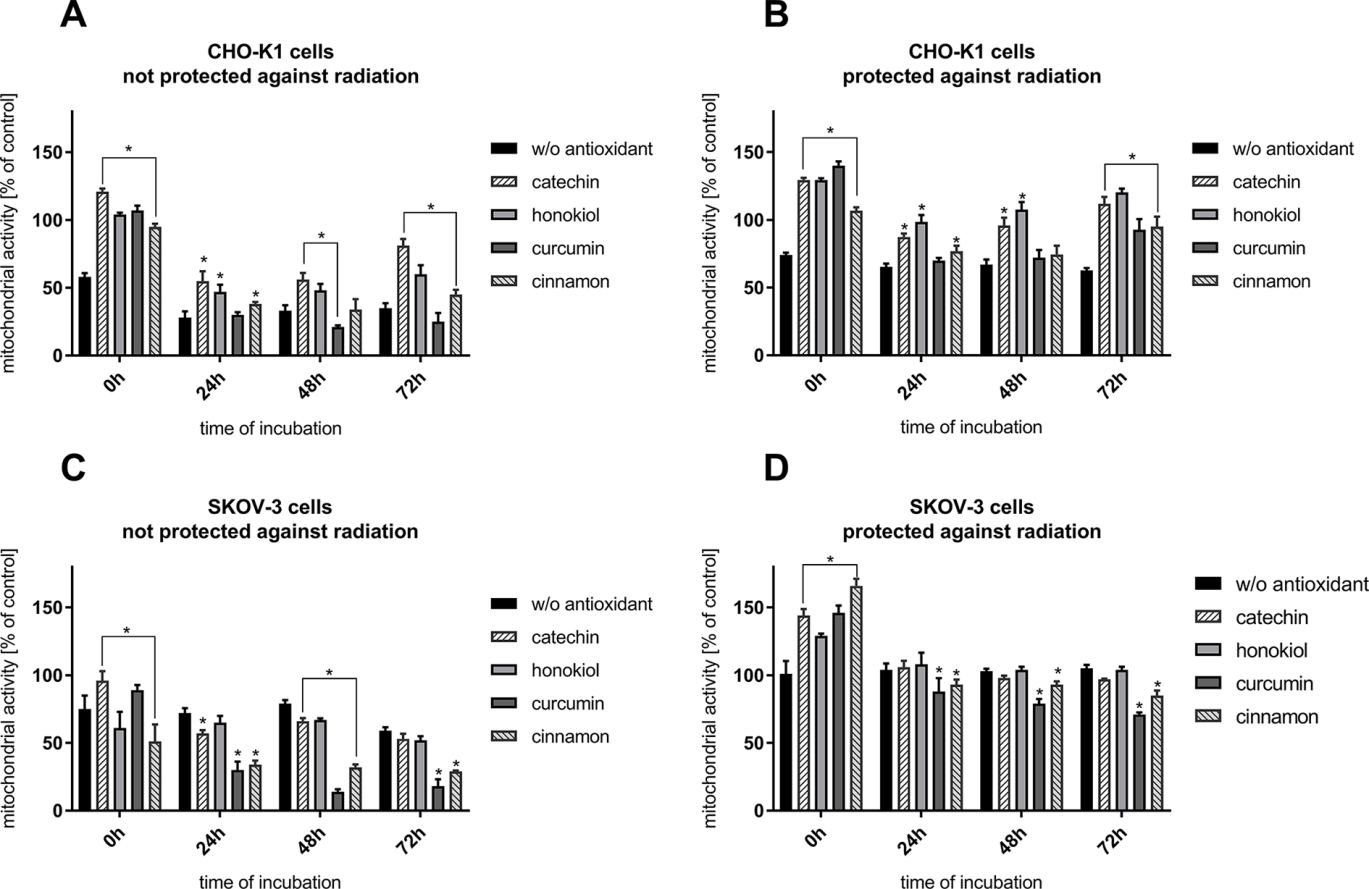
Cells viability verified by MTT assay after the balloon flight. **(A)** CHO-K1 cells not protected against radiation, **(B)** CHO-K1 cells protected by the aluminum foil against radiation, **(C)** SKOV-3 cells not protected against radiation, **(D)** SKOV-3 cells protected by aluminum foil against radiation (*p ≤ 0.05). Data are presented as the mean percentage relative to control cells, which were not treated with antioxidants and not sent into the stratosphere.

Following the flight, we observed a decrease in the mitochondrial activity of CHO-K1 and SKOV-3 cells not protected against radiation and not incubated with antioxidants (**Figures 2A, C**). The presence of antioxidants, except for curcumin, caused the increase in the CHO-K1 cells’ viability. The most protective effect of antioxidants was observed in CHO-K1 cells incubated with catechin and honokiol. Studies have shown a slight increase in mitochondrial activity in normal cells after treating with cinnamon. Curcumin was the most lethal for these cells (72 h–25%) (**Figure 2A**). A significant increase in the mitochondrial activity in CHO-K1 cells protected against radiation was additionally observed, especially after catechin and honokiol treatment (**Figure 2B**).

The results have shown that SKOV-3 cells not protected against radiation display similar mitochondrial activity after balloon flight —∼70% in comparison to CHO-K1 cells (**Figure 2C**). Furthermore, it was observed that curcumin and cinnamon were the most lethal for SKOV-3 cells, as their mitochondrial curcumin and cinnamon activity was decreasing repetitively approximately to the 18 and 30%. Other substances used in this experiment exert less lethal effect on SKOV-3 cell line. The most protective antioxidant for the cells was catechin. However, there was no increase in mitochondrial activity in SKOV-3 cells incubated with honokiol. It was observed that curcumin significantly diminished mitochondrial metabolism in both cell lines.

The mitochondrial activity of cancer cells incubated without antioxidants and protected against radiation remained unchanged (∼100%) in all 72 h (**Figure 2D**). Directly after the flight, we observed the highest mitochondrial activity when the antioxidants were applied. A significant decrease was observed after usage of curcumin—88% after 24 h, 79% after 48 h, and 71% after 72 h. However, a smaller yet still significant drop was observed after cells’ incubation with cinnamon. Catechin and honokiol application generated results similar to the control cells and cells without antioxidants.

### Cell Death

**Figure 3:**
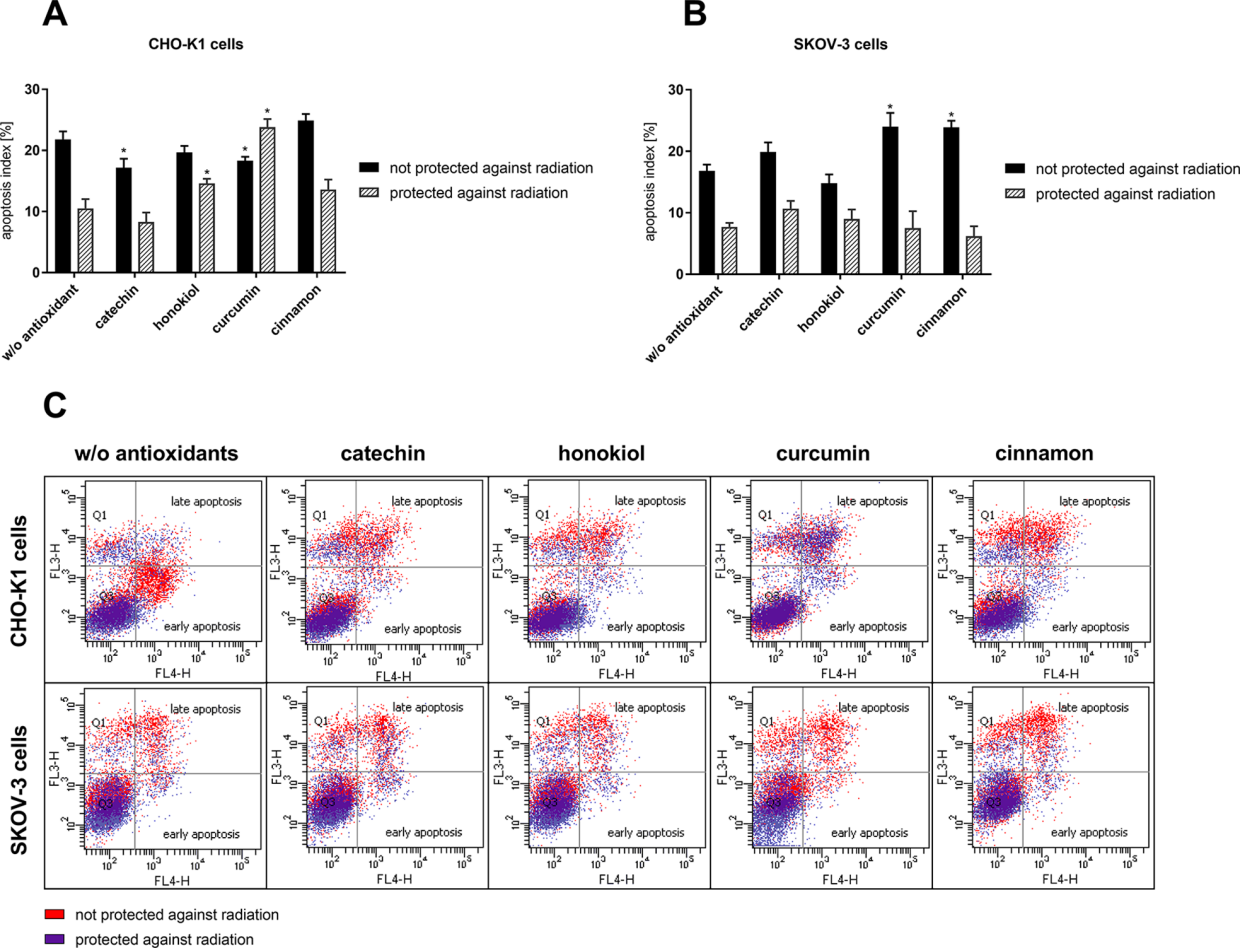
The percentage of apoptotic cells evaluated by Annexin V-APC/PI staining 24 h after balloon flight. **(A)** CHO-K1 and **(B)** SKOV-3 cells protected by aluminum foil and not protected against radiation (*p ≤ 0.05; data are presented as the mean percentage of measured apoptotic cells); **(C)** the comparison of early and late apoptotic CHO-K1 and SKOV-3 cells after 24 h incubation after the balloon flight; results are visualized as representative dot plots.

Cell death assay revealed different values of apoptotic and necrotic cells after the balloon flight. We noticed the increased percentage of necrotic cells in case of CHO-K1 cells exposed to radiation in comparison to CHO-K1 cells protected against radiation (**Figure 3**, **Table 1**). Incubation with catechin, honokiol, or curcumin resulted in a reduced level of total apoptotic cells, whereas cinnamon has been revealed to be the strongest proapoptotic agent.

**Table 1:** Cell death evaluated by Annexin V-APC/PI staining of CHO-K1 and SKOV-3 cells protected and not protected against radiation, treated with various antioxidants, after 24 h incubation after balloon flight. Results expressed as percentage of control cells which were not treated with antioxidants and not sent into the stratosphere.

CHO-K1 cells	Undamaged	Necrotic	Early apoptotic	Late apoptotic		SKOV-3 cells	Undamaged	Necrotic	Early apoptotic	Late apoptotic
***Not protected against radiation***											***Not protected against radiation***				
**W/o antioxidant**	66.7 ± 4.34	11.5 ± 1.32	5 ± 0.42	16.8 ± 0.91		**W/o antioxidant**	75.2 ± 4.67	8.0 ± 0.43	2.9 ± 0.16	13.9 ± 0.64
**Catechin**	72.6 ± 5.24	10.2 ± 1.02	2.5 ± 0.12*	14.7 ± 0.57*		**Catechin**	70.9 ± 5.83	9.2 ± 0.46	4.3 ± 0.18*	15.6 ± 0.78*
**Honokiol**	71.3 ± 4.52	9.0 ± 0.67*	3.7 ± 0.13*	16 ± 0.46		**Honokiol**	76.5 ± 5.73	8.7 ± 0.32	2.5 ± 0.13	12.3 ± 0.61*
**Curcumin**	72.2 ± 5.87	9.5 ± 0.87	1.5 ± 0.09*	16.8 ± 0.65		**Curcumin**	64.1 ± 2.90*	11.9 ± 0.97	6.3 ± 0.21*	17.7 ± 0.81*
**Cinnamon**	61.2 ± 3.21	13.9 ± 1.91*	2.5 ± 0.11*	22.4 ± 0.14*		**Cinnamon**	65.0 ± 3.74*	11.1 ± 0.56	3.8 ± 0.22*	20.1 ± 0.89*
***Protected against radiation***						***Protected*** ***against radiation***				
**W/o antioxidant**	82.6 ± 4.32	6.9 ± 0.43	6.6 ± 0.32	3.9 ± 0.2		**W/o antioxidant**	88.9 ± 5.55	3.4 ± 0.12	3.9 ± 0.19	3.8 ± 0.19
**Catechin**	84.3 ± 3.21	8.0 ± 0.87	3.8 ± 0.09*	4.5 ± 0.23		**Catechin**	85.0 ± 4.98	4.3 ± 0.23	5.4 ± 0.27*	5.3 ± 0.21*
**Honokiol**	79.9 ± 3.58	5.0 ± 0.32	9.7 ± 0.32*	4.9 ± 0.25*		**Honokiol**	85.7 ± 4.67	5.3 ± 0.19	4.4 ± 0.24*	4.6 ± 0.21*
**Curcumin**	68.9 ± 4.01*	7.3 ± 0. 58	7.1 ± 0.29	16.7 ± 0.59*		**Curcumin**	87.3 ± 4.77	5.3 ± 0.57	3.9 ± 0.22	3.6 ± 0.09
**Cinnamon**	77.3 ± 4.63	9.1 ± 0.21	8.9 ± 0.42*	4.7 ± 0.48		**Cinnamon**	90.0 ± 3.42	3.8 ± 0.09	3.3 ± 0.18*	2.9 ± 0.14*

*p ≤ 0.05.

The highest percentage of late apoptotic cells was observed after curcumin treatment of CHO-K1 cells protected against radiation. Interestingly, incubation with honokiol and cinnamon resulted in the increased level of early apoptotic cells. The lowest level of total apoptotic cells was observed after the catechin treatment.

SKOV-3 cells not protected against radiation showed the increased percentage of apoptotic cells in comparison to the cells protected against radiation. The highest level of dead cells was observed after treatment with curcumin and cinnamon, whereas honokiol exhibited antiapoptotic properties.

In the case of SKOV-3 cells protected against radiation, similar percentage of apoptotic cells, for the cells both incubated with and without antioxidants, was observed. The highest level of necrotic cells was revealed for the cells treated with honokiol and curcumin. However, the reduced percentage of apoptotic cells was detected only for the cells incubated with cinnamon.

### Membrane Permeabilization

**Figure 4:**
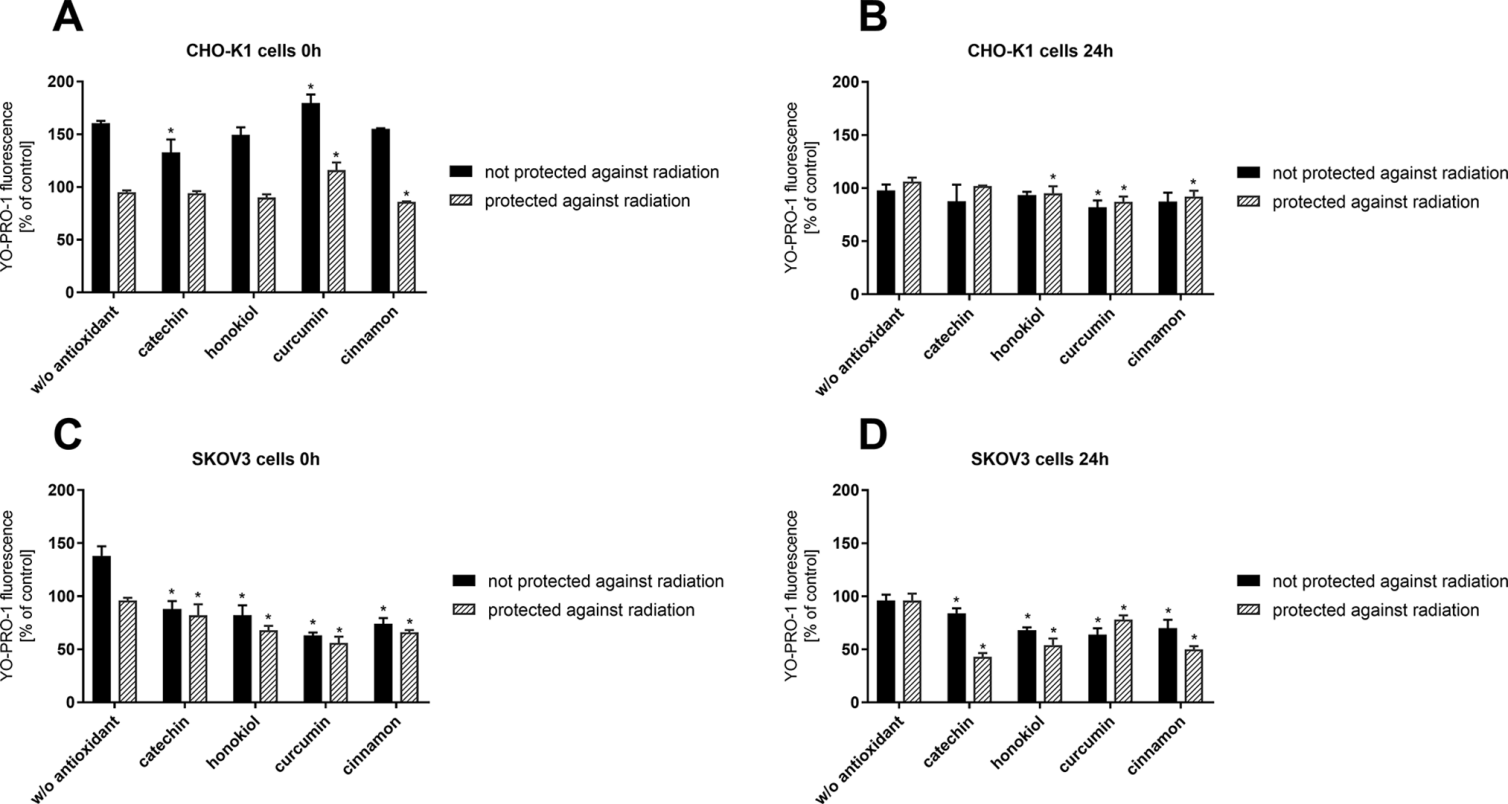
Cell membrane permeabilization determined by YO-PRO-1 fluorescence after the balloon flight. CHO-K1 cells directly after the flight **(A)** and 24 h incubation **(B)** and SKOV-3 cells directly after the flight **(C)** and 24 h incubation **(D)** (*p ≤ 0.05). Data are presented as the mean percentage relative to control cells, which were not treated with antioxidants and not sent into the stratosphere.

Reduced membrane permeabilization is crucial due to the greater cell protection against harmful radiation and temperature shock. Directly after the stratospheric balloon flight (0 h), high membrane permeabilization in CHO-K1 cells not protected against radiation was observed (**Figure 4**). After 24 h, the membrane permeabilization for all samples decreased significantly. However, it was observed that the cells after preincubation with catechin have less permeable cell membranes when compared to the other samples. In the case of CHO-K1 cells not protected against radiation, a constant level of cell membrane permeabilization regardless of the type of antioxidant was observed.

Our studies indicated a decreased membrane permeabilization in SKOV-3 cells not protected against radiation and incubated with antioxidants. The same dependency was observed in the case of SKOV-3 cells protected against UV radiation. Of all the tested compounds, once again, catechin showed the most protective effect due to the reduced permeability of the plasma membrane.

### Intracellular Reactive Oxygen Species (ROS) Generation

**Figure 5:**
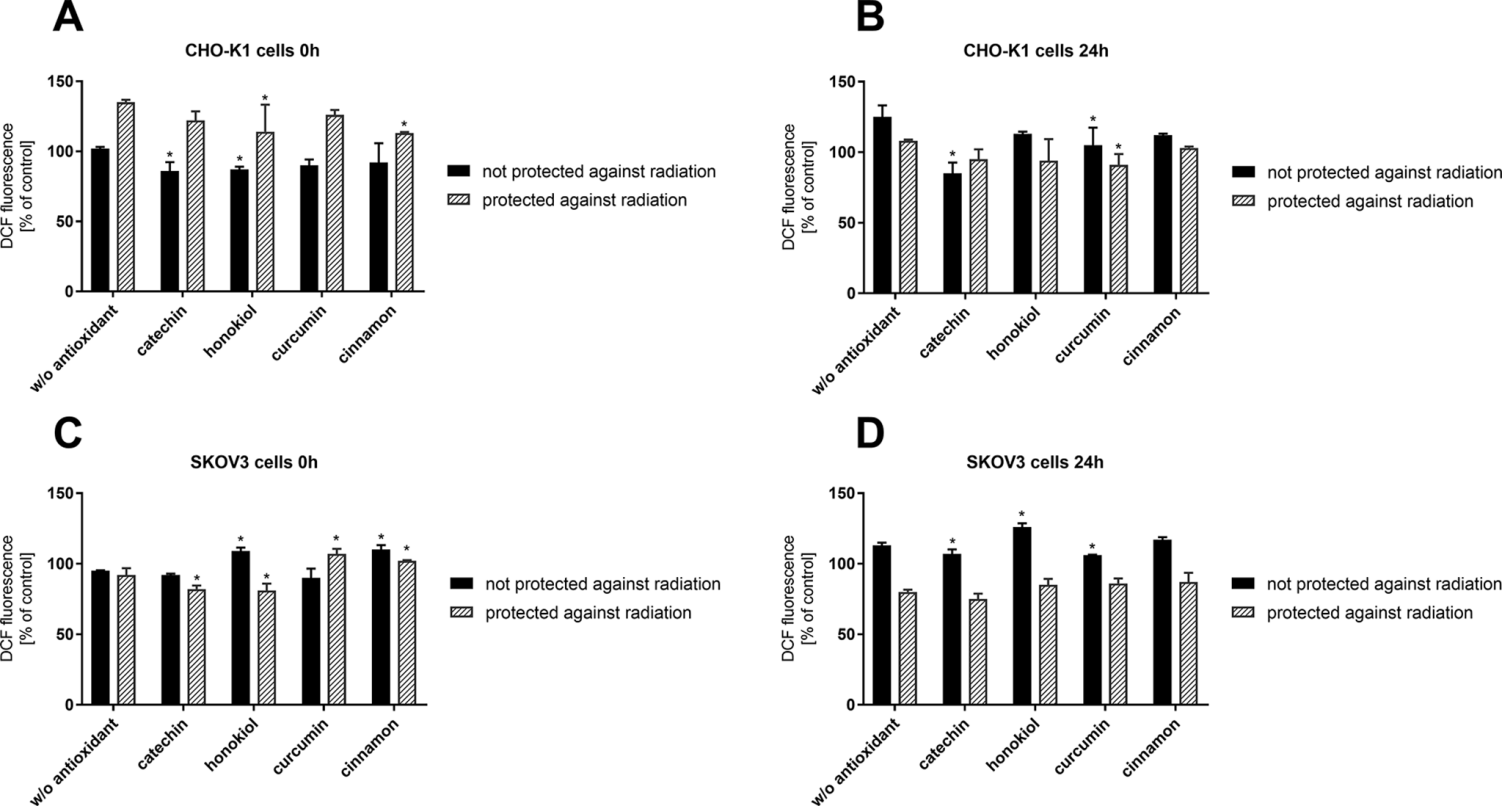
Intracellular reactive oxygen species generation verified by DCF assay after the balloon flight. CHO-K1 cells directly after the flight **(A)** and 24 h incubation **(B)** and SKOV-3 cells directly after the flight **(C)** and 24 h incubation **(D)** (*p ≤ 0.05). Data are presented as the mean percentage relative to control cells, which were not treated with antioxidants and not sent into the stratosphere.

Directly after the stratospheric flight (0 h), a significant decrease in intracellular ROS generation in CHO-K1 cells not protected against radiation in the case of pretreatment with catechin and honokiol was observed (**Figure 5**). However, after 24 h, an increased level of ROS was detected. In the case of CHO-K1 not protected against radiation and pretreated with catechin, the lowest and relatively constant level of ROS was detected. Those results confirmed that catechin displays the most valuable protective role among all of the tested compounds.

Furthermore, directly after the balloon flight and 24 h incubation, there was a reduced level of oxidative stress after pretreatment with various antioxidants in CHO-K1 cells protected against radiation measured. However, it was still similar to the cells incubated in antioxidant-free medium.

Moreover, our studies revealed that in SKOV-3 cells not protected against radiation, the level of oxidative stress after pretreatment with curcumin and catechin was almost the same as in the cells incubated without antioxidant. Treatment with honokiol and cinnamon resulted in the increase in ROS generation.

In the case of SKOV-3 cells protected against radiation, a higher level of ROS in cells incubated with curcumin and cinnamon was observed. The results indicated that those compounds could display antitumor activity by generating ROS in cancer cells, which was reflected in our research. After 24 h, the level of oxidative stress for all the tested compounds and cells without antioxidant was at 80%.

In summary, our results have shown that intracellular ROS generation is related to the exposure to stratospheric conditions, especially radiation, and treatment with the examined antioxidants, which act differently in various cells.

### SOD2 Expression

**Figure 6:**
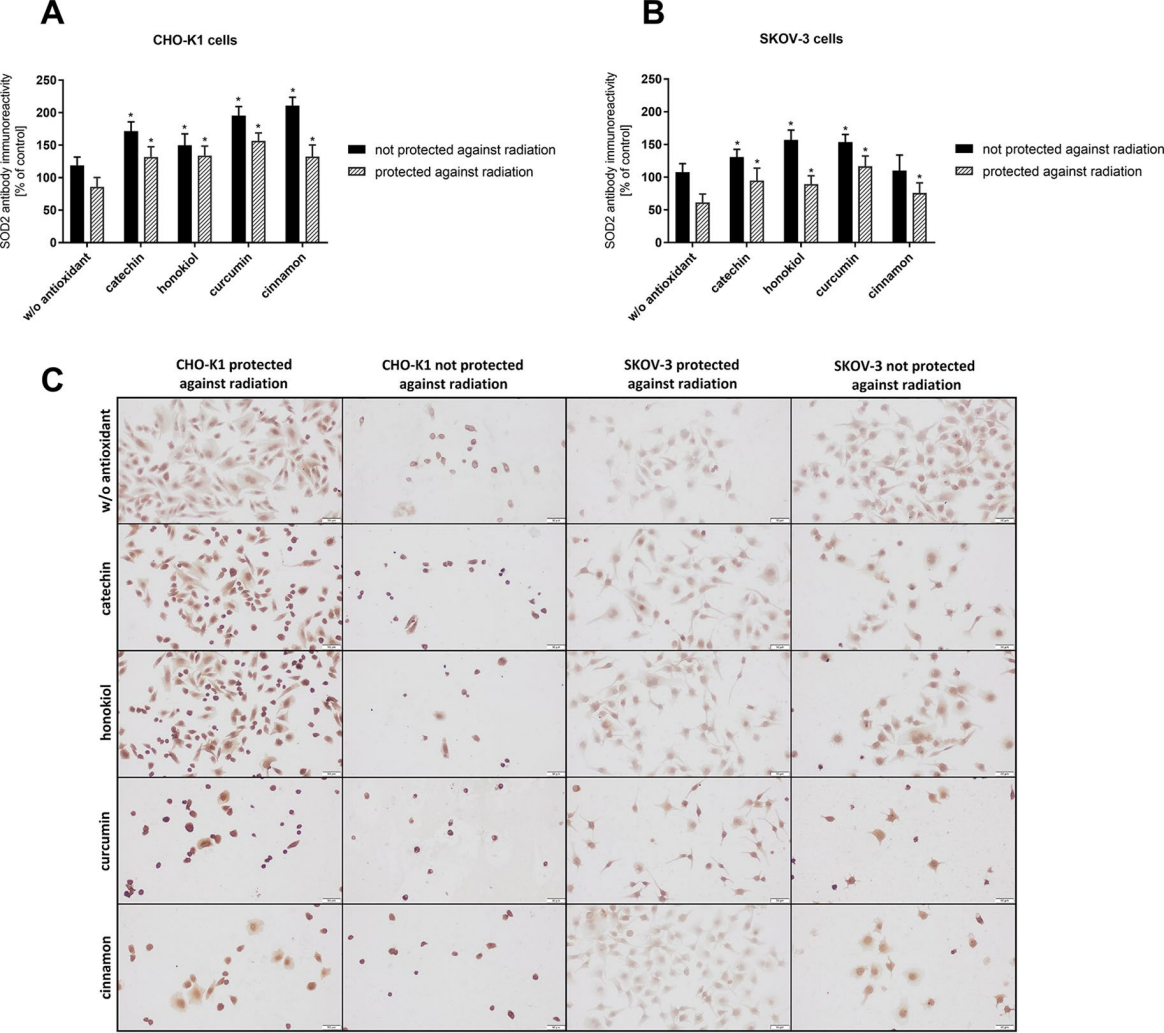
The mean intensity of immunocytochemical reaction with SOD2 antibody in CHO-K1 and SKOV-3 cell lines after the balloon flight in the presence of various compounds, taking into account the exposure and the protection against radiation. **(A)** CHO-K1 and **(B)** SKOV-3 cells protected by aluminum foil and not protected against radiation (*p ≤ 0.05; data are presented as the mean percentage relative to control cells which were not treated with antioxidants and not sent into the stratosphere); **(C)** the representative microphotographs of the immunoreactivity of SOD2 antibody in CHO-K1 and SKOV-3 cells protected and not protected against radiation.

Immunocytochemical staining method enabled detection of the significant differences in SOD2 expression after the balloon flight in both examined cell lines (**Figure 6**). After the stratospheric flight, we observed an increased expression of the manganese-dependent superoxide dismutase in CHO-K1 cells in comparison to SKOV-3 cells in the presence of catechin, curcumin, and cinnamon extract. CHO-K1 cells not protected against radiation and treated with curcumin and cinnamon extract were the most immunoreactive. In the case of SKOV-3 cells, the highest expression of SOD2 was revealed when the cells were treated with honokiol and curcumin. The obtained results indicate that the examined antioxidants induced SOD2 activity in normal cells more effectively than in cancer cells.

### Proliferation

**Figure 7:**
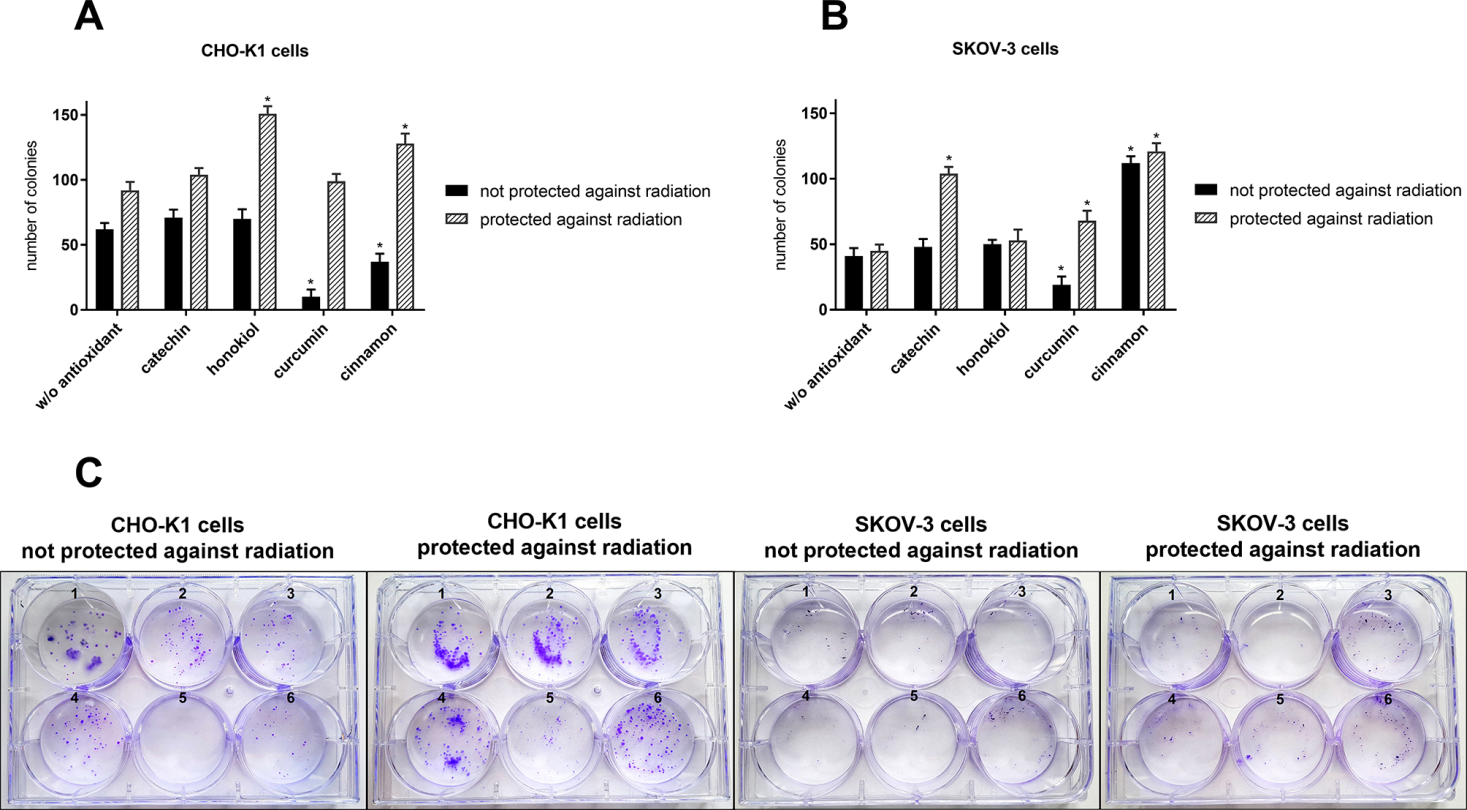
Number of colonies after the balloon flight and 7-day incubation of **(A)** CHO-K1 and **(B)** SKOV-3 cells protected by aluminum foil and not protected against radiation in the presence of various compounds (*p ≤ 0.05; data are presented as the mean amount of counted colonies); **(C)** the representative pictures of colonies: 1, control cells which were not treated with antioxidants and not sent into the stratosphere; 2, w/o antioxidant; 3, catechin; 4, honokiol; 5, curcumin; and 6, cinnamon.

The clonogenic assay confirmed the protective role of selected antioxidants, which enhanced cell survival after exposure to the stratospheric environment (**Figure 7**). Differences in the number of colonies between the protected and unprotected cells indicate the cytotoxic properties of radiation. Furthermore, this assay confirmed that CHO-K1 was more sensitive to high energy particles in the stratosphere. We observed more and bigger colonies in CHO-K1 cells, especially among the protected cells. Curcumin was the least efficient in cell protection, whereas the highest number of colonies was observed after cinnamon and catechin pretreatment.

A comparison of MTT, cell death, and clonogenic assay for SKOV-3 cells not protected against radiation and treated with cinnamon revealed a decrease in cell viability after the balloon flight; however, proliferation ability was not affected. It suggests that cinnamon may first increase cell death after the flight and then promote cells’ multiplying. We did not observe this effect for curcumin treatment.

### DNA Damage in the Stratosphere

**Figure 8:**
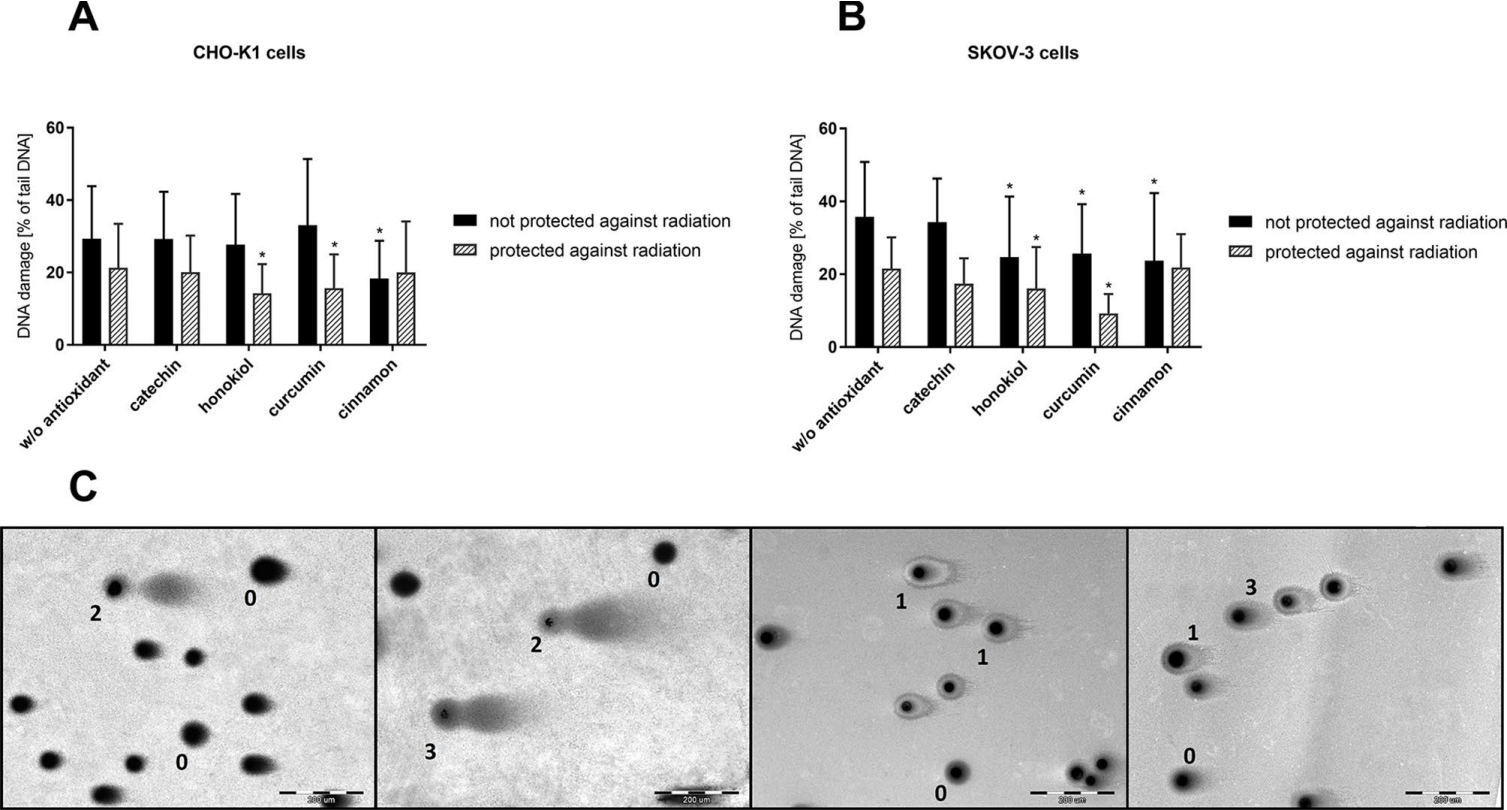
DNA damage evaluation of CHO-K1 and SKOV-3 cells protected and not protected against radiation, treated with various antioxidants, directly after balloon flight. **(A)** CHO-K1 and **(B)** SKOV-3 cells protected by aluminum foil and not protected against radiation (*p ≤ 0.05; data are presented as percentage of damaged DNA presented in tail of comet); **(C)** different types of DNA damage evaluated by comet assay; type 0, size of the head the normal nucleus size, tail absent; type 1, size of the head the normal nucleus size, tail size less than normal nucleus size; type 2, size of the head less than half size of normal nucleus, tail size more than two times the normal nucleus size; type 3, size of the head the normal nucleus size, tail size about two times the normal nucleus size.

In neutral comet assay, we were able to distinguish four types of comets. **Figure 8** shows the detailed categorization method used for the evaluation of our samples that allows us to divide the observed nuclei into three groups: undamaged, apoptotic, and intermediately damaged (**Table 2**). A comparison of CHO-K1 and SKOV-3 cells not protected against radiation revealed protective role of antioxidants in DNA damage among CHO-K1 cells; however, curcumin and cinnamon promoted DNA destruction in SKOV-3 cells. In the cells protected against radiation, antioxidants acted differently. In normal cells, we observed more undamaged cells after preincubation with catechin and honokiol, whereas in cancer cells, cinnamon additionally reduced the percentage of cells with DNA damages. These results suggest that the protective role of antioxidants in DNA damages depends on the presence of radiation resulting in various activity of these substances in cells.

**Table 2:** DNA damage evaluation using comet assay of CHO-K1 and SKOV-3 cells protected and not protected against radiation, treated with various antioxidants, directly after balloon flight. Results expressed as percentage of control cells which were not treated with antioxidants and not sent into the stratosphere.

CHO-K1 cells	Undamaged	Apoptotic damaged	Intermediately damaged	SKOV-3 cells		Undamaged	Apoptotic damaged	Intermediately damaged
***Not protected against radiation***					***Not protected against radiation***			
**W/o antioxidant**	79.2 ± 3.64	8.85 ± 0.42	11.95 ± 0.74		**W/o antioxidant**	71.17 ± 4.62	0.9 ± 0.04	27.93 ± 2.58
**Catechin**	83.79 ± 4.52	3.16 ± 0.12*	13.04 ± 1.05		**Catechin**	81.64 ± 1.41*	0.66 ± 0.02*	17.7 ± 0.95*
**Honokiol**	84.12 ± 3.74	1.01 ± 0.09*	14.86 ± 1.23*		**Honokiol**	83.33 ± 3.46*	0.6 ± 0.03*	16.07 ± 0.45*
**Curcumin**	90.58 ± 6.74	0.65 ± 0.03*	8.77 ± 0.63*		**Curcumin**	63.7 ± 2.94	0.0 ± 0.0*	36.3 ± 3.85*
**Cinnamon**	83.04 ± 5.97	6.57 ± 0.29*	10.38 ± 0.23		**Cinnamon**	61.04 ± 2.96*	0.97 ± 0.06	37.99 ± 3.94*
***Protected against radiation***					***Protected against radiation***			
**W/o antioxidant**	89.75 ± 5.62	2.05 ± 0.11	8.2 ± 0.42		**W/o antioxidant**	53.77 ± 4.31	1.26 ± 0.09	44.98 ± 4.02
**Catechin**	91.36 ± 7.32	0.0 ± 0.0*	8.64 ± 0.22		**Catechin**	74.46 ± 3.62*	0.43 ± 0.07*	25.11 ± 1.86*
**Honokiol**	92.71 ± 6.62	2.08 ± 0.32	5.21 ± 0.24*		**Honokiol**	82.77 ± 2.47*	1.13 ± 0.16	16.1 ± 0.95*
**Curcumin**	82.83 ± 6.02	0.43 ± 0.02*	16.74 ± 1.45*		**Curcumin**	51.99 ± 3.38	0.25 ± 0.04*	47.76 ± 3.79
**Cinnamon**	89.29 ± 5.83	2.38 ± 0.16	8.33 ± 0.78		**Cinnamon**	69.81 ± 5.03*	0.97 ± 0.05*	29.22 ± 2.05*

Furthermore, using the CometScore software, we were able to perform the analysis of the pictures of comets and determine the percentage of the damaged DNA accompanying the flight. Our studies showed that cancer cells were more vulnerable to UV radiation exposure causing an increase in the presence of DNA in the tail. The most active compound preventing DNA damage was cinnamon extract, both for normal and cancer cells. In the case of protected cells, honokiol and curcumin allowed for the most efficient preservation.

### Confocal Microscopy

**Figure 9:**
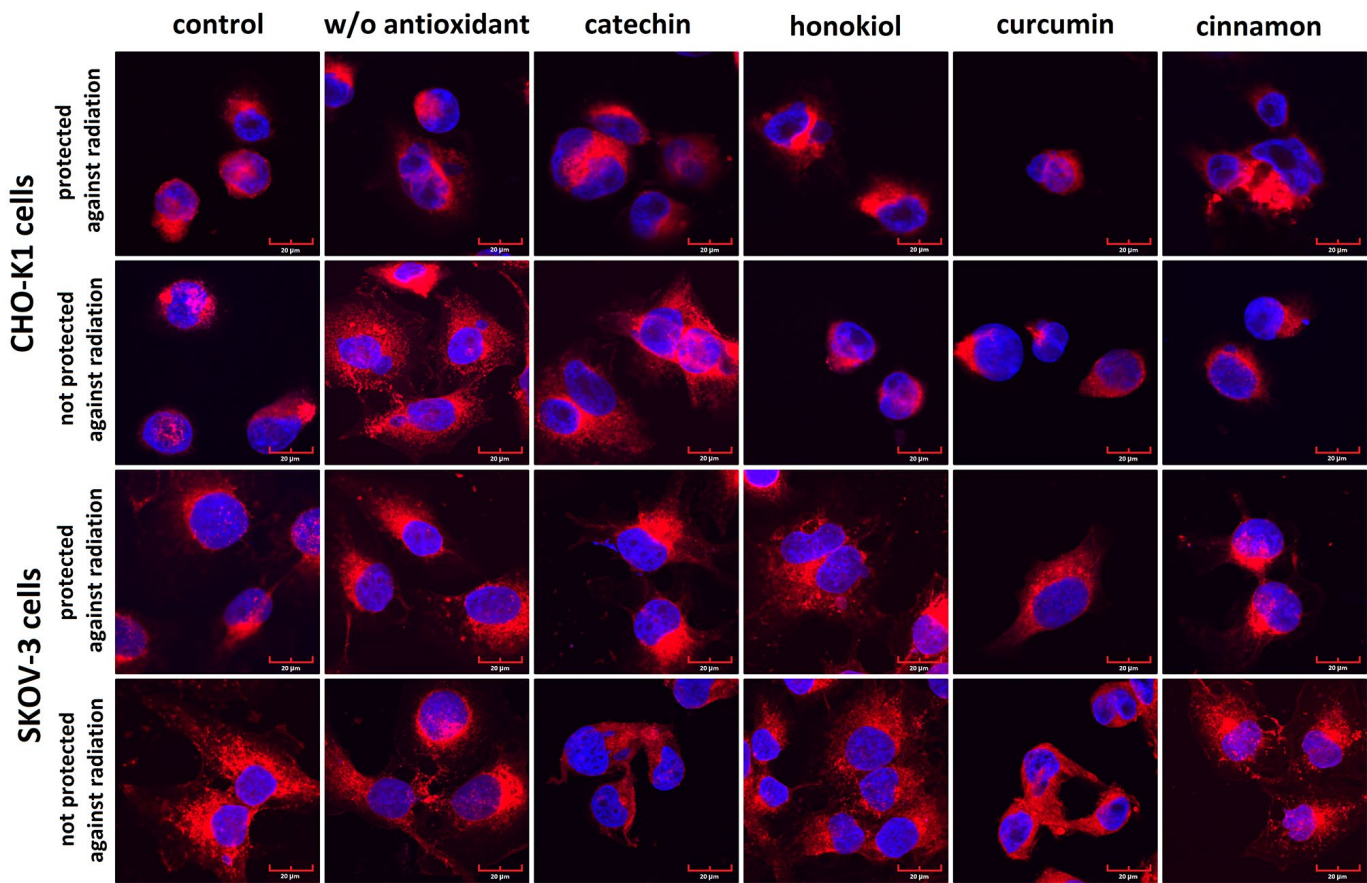
The representative photographs of the morphology of CHO-K1 and SKOV-3 cells protected and not protected against radiation stained with CellMask™ Deep Red (cell membranes) and DAPI (nuclei).

Fluorescence staining revealed slight differences in the cell morphology after the balloon flight in both examined cell lines (**Figure 9**). After the stratospheric flight, we observed more slender projections in both cell lines. CHO-K1 and SKOV-3 cells treated with honokiol, curcumin, and cinnamon extract were morphologically similar to the control cells. The cells exposed to radiation were enlarged, especially CHO-K1 cells incubated without antioxidant and protected against radiation. Some nuclei were fragmented, in particular in the cells not protected against radiation. However, the incubation with an antioxidant resulted in an increased percentage of cells with condensed nuclei containing many nucleoli.

## Discussion

During balloon flight, the cell samples were exposed to several fluctuating stressful factors, namely, radiation, temperature, pressure, and overload. However, as long as the exposure to these factors does not reach extreme levels, most of the cells are still able to survive due to their natural capability of activating a variety of specific defensive pathways. The stress response could be dramatically different among various types of cells, notably normal and cancer cells. Therefore, application of meticulously selected compounds could either alleviate or exacerbate the consequences of the exposure to rapidly varying temperature, pressure, and UV conditions during stratospheric balloon campaign—depending on their various properties and type of cells—hence influencing not only the survival but also recovery of cells after the experiment. Natural-derived substances, such as catechin, curcumin, cinnamon extract, and honokiol have been recently gaining much attention as therapeutic compounds for cancer prevention and treatment. Our study represents the first attempt at identifying compounds exhibiting protective properties in space by launching human normal and cancer cells into the stratosphere on a meteorological balloon.

There is a huge ambiguity among the available data concerning quantification of the absorbed dose of cosmic rays depending on the length of the flight and height reached during the meteorological balloon mission. The intensity of the radiation varies depending on parameters such as location and time unit, latitude and longitude, ozone depth, and solar activity (Mertens et al., 2012). The average dose of ionizing radiation reaching the atmosphere is estimated at around 2–20 μSv (Mertens et al., 2012). In the stratosphere, 30,000 m above the Earth’s surface, the UV radiation is much higher than on the sea level (Cadet et al., 2005). Exposure to radiation is closely related to the thickness of the atmosphere, and with increasing altitude, the protective layer of the atmosphere becomes thinner; thus, the radiation doses are higher (Lim and Bagshaw, 2002). During the flight, the cells were deprived of a protective effect of the ozonosphere and troposphere (which naturally protect Earth against radiation), resulting in decreased cell viability after the flight.

During the experiment, half of the samples were covered with aluminum foil. Therefore, we were able to evaluate the effect of radiation (mainly UV and high energy particles such as α, β^−^) on examined cells in the presence of various compounds. We observed increased mitochondrial activity and decreased level of apoptotic cells after pretreatment with various antioxidants, especially among CHO-K1 cells. Catechin was the most efficient in radioprotection of normal cells, whereas honokiol appeared to be the most protective for cancer cells. These results were reflected by intracellular ROS measurements and comet assay. We did not observe significant differences in DCF fluorescent signal between protected and unprotected SKOV-3 cells. However, in cell death assay and immunocytochemical staining, the results were altered. It could indicate that radiation-related death in SKOV-3 cells was not strictly related to the oxidative stress and caused direct cell damage.

According to the time of cell death after irradiation, two types of apoptosis can be distinguished: early apoptosis occurring straight after G2 arrest and late apoptosis, when cell death is preceded by one or more cell divisions. Widely understood radiosensitivity has been demonstrated to vary among different cell types or even populations (Cohen-Jonathan et al., 1999). For instance, thymocytes (Yamaguchi, 1967) or lymphocytes (Yamaguchi, 1967) exposed to radiation were early directed to the apoptotic pathway, whereas Yanagihara et al. (1995) described human gastric epithelial tumor cells exhibiting delayed radiation-induced apoptosis, which reached maximum after 72–96 h (Yanagihara et al., 1995). We observed a higher percentage ratio of apoptotic cells among normal cells (CHO-K1) as compared to cancer cells (SKOV-3). Our studies suggest that normal cells are more sensitive to UV radiation in stratosphere, which resulted in earlier and more intensive apoptosis among CHO-K1 cells. The significant differences were also shown in response to different antioxidants of each cell type, which should be highlighted as particularly interesting in the case of curcumin, which acted as light sensitizer and induced cell death more effectively in SKOV-3. Similar results were observed after treatment with catechin—we revealed higher percentage ratio of early apoptotic cells among SKOV-3 cells exposed to radiation than among CHO-K1 cells cultured with the green tea polyphenol (4.3 vs. 2.5%). Furthermore, we noticed less and smaller SKOV-3 colonies in comparison to CHO-K1 in clonogenic assay, which indicated a delayed radiation-induced death of cancer cells. In addition, performed tests highlighted the considerable impact of honokiol and catechin on recovery of the cells after the flight, proving cytoprotective role of antioxidant after radiation exposure.

Radiation-related DNA strand breaks, confirmed in comet assay, have been demonstrated to initiate the expression of p53 (Mihara et al., 2003), which promotes cytochrome C release from the mitochondria (Hosoki et al., 2012) and activation of caspase cascade leading to apoptosis (Garland and Rudin, 1998). SOD2 is the essential mitochondrial antioxidant enzyme which plays a crucial role in protection against radiation in cells (Hirose et al., 1993; Epperly et al., 1999) by scavenging the free radical superoxide (Candas and Li, 2014). Overexpression of SOD2 stabilizes mitochondrial membrane and protects complexes I and III of the respiratory chain from radiation-induced damages (Pearce et al., 2001), as well as decreasing the release of cytochrome C, resulting in the apoptosis blockage (Epperly et al., 2009). Owing to the intranuclear localization of the *SOD2* gene, SOD2 is synthesized in the cytoplasm and subsequently imported into mitochondria *via* the mitochondrial protein influx (MPI), where a manganese ion is combined with SOD2 to activate the enzyme (Wispé et al., 1989). The research conducted on mammalian cells has demonstrated a decrease in the mitochondrial protein import through MPI caused by radiation, which leads to the accumulation of precursor proteins outside mitochondria degraded by proteasomes (Wright et al., 2001) and the reduced number of mitochondrial proteins. Furthermore, Azzam et al. (2012) revealed that MPI proteins may be damaged by irradiation, resulting in deficient protein import (Azzam et al., 2012). Accordingly, mitochondria play a critical role in radioprotection by affecting SOD2 activity through Cdk1-p53-mediated SOD2 regulation. Transcriptional factors promoted by reduction in ROS production induce transcription of SOD2-regulated genes triggering adaptive response to the oxidative stress. Thus, substances that increase SOD2 activity enhance stability of the mitochondrial membrane and block the activation of apoptosis. Natural derivatives used in our study have been displayed as radioprotective substances (Goel and Aggarwal, 2010; Chilampalli et al., 2010; Sevgi et al., 2015) promoting SOD2 activity (Evacuasiany et al., 2014; Khristi et al., 2014; Zhai et al., 2015; Huang et al., 2017). Our studies revealed that the amount of SOD2 is significantly increased in normal cells treated with the tested antioxidants, whereas we observed a significantly increased levels of ROS among cancer cells. In addition, cancer cells have exhibited lower viability compared to normal cells.

Overall, curcumin occurred to be the most efficient radioprotective substance in normal cells while acting conversely in SKOV-3. That particular phenomenon of curcumins’ selective cytotoxicity has already attracted widespread interest to be applied in cancer therapy (Aggarwal et al., 2013; Sharma et al., 2004; Hsu and Cheng, 2007; Dhillon et al., 2008) and seems to be a promising protective agent. Notably, photocytotoxic activity of curcumin in cancer cells, extensively described in the literature (Verwanger et al., 2011; Banerjee et al., 2012; Duse et al., 2018; Khorsandi et al., 2019; Machado et al., 2019; Roos et al., 2019), was also observed in our study. Photodynamic effect of curcumin is associated with increased ROS production and generation of singlet oxygen after photoexcitation triggered by UV light exposure (Priyadarsini, 2009; Sarkar and Hussain, 2016; Duse et al., 2018).

According to the data recorded by on-board sensors, the temperature varied between −36 and 20°C. Lower cultivation temperature contributed to a decreased cellular metabolism (Ducommun et al., 2002), projecting on prolonged preservation of cell viability. However, moderately cold temperature conditions (−60 to −15°C) remain the main limiting factor affecting the cell viability (Gao and Critser, 2000). During the flight, the biological samples were exposed to this stressful temperature for 2 h and experienced repeated cycle of chilling and warming while the balloon was ascending and descending (**Figure 1**): first, when temperature dropped down to −21°C, second when it raised to −2°C, and third when it dropped down to −36°C before raising to 20°C in the landing area. However, the air density in the stratosphere is ∼1,000 times lower than on sea level, and because of that, the temperature exchange between the biological samples and air was limited, resulting in the relative temperature stability of cells. Along with extensive structural damage of the cell, freezing and thawing cycles are associated with oxidative stress and consequently mitochondrial disturbance, cell membrane permeabilization, or DNA damage (Karimi-busheri et al., 2016), finally leading to death, triggering either apoptotic or necrotic pathway (Baust et al., 2009; Savitskaya and Onishchenko, 2016), which was reflected in our research.

The biological samples after the balloon flight exhibited an increase in ROS generation and percentage of dead cells; however, treatment with the selected antioxidants limited these processes. Antioxidants are known to alter the properties of cell membranes, e.g., plant flavonoids are incorporated into hydrophilic area of the membrane which changes its physical properties (Cyboran et al., 2012) and may affect lipid rafts, cellular metabolism, and cell signaling pathways (Tarahovsky et al., 2014); more hydrophilic flavonoids react with the polar head groups of membrane lipids preserving functions and structures of membranes (Oteiza et al., 2005), whereas isoflavones decrease cell membrane fluidity in cancer cells (Ajdžanović et al., 2013). Curcumin has been proven to have similar properties. The compound affects membrane structure (Barry et al., 2009) by incorporation into lipid bilayer (Ben-Zichri et al., 2019), modulates the lipid raft domains (Tsukamoto et al., 2014), affects the lipid membrane making it thinner, and weakens its elasticity (Hung et al., 2008). According to these findings, the tested antioxidants may have altered the physical properties of cell membrane resulting in its increased stability in lower temperature and decreased permeabilization, which was measured in YO-PRO-1 assay directly after the balloon flight and highlighted in CLSM analysis. In this process, catechin was the most efficient in CHO-K1 cells. However, we noticed that membrane permeability increased after incubation with curcumin in CHO-K1 cells not protected against radiation after the flight, whereas it was strongly reduced in SKOV-3 cells exposed to the stratospheric conditions.

A number of studies suggest that cryopreservation and low temperature affect the cell sensitivity to radiation (Cugia et al., 2011). Accordingly, the data concerning utilization of frozen Chinese hamster fibroblasts revealed the decrease in X-ray-induced damage of cells stored at −196°C in comparison to cells irradiated at room temperature (Ashwood-Smith and Friedmann, 1979). Information from cryocrystallography demonstrates that extremely low temperatures (−173°C) stop the diffusion of free radicals caused by irradiation, which leads to less harmful cell damages induced by X-rays (Garman, 2003). Owing to this fact, the increased survival of cells affected by freezing may be associated with reduced oxidative stress and damage at low temperature (Cugia et al., 2011). Altogether, the data suggest that freezing can protect cells from radiation-induced damage and apoptosis, affecting cell viability after the flight.

There is a huge ambiguity between the response to the temperature-related stress in different cell types. Our experiment confirms that mechanisms of cellular responses to oxidative stress are strongly associated with the type of cell. In general, treatment with antioxidants resulted in an increased viability in normal cells, whereas oxidative stress was intensified in cancer cells. However, the SOD2 expression was higher in normal cells. It shows that molecular antioxidative mechanism of different antioxidants varies in diverse types of cells: the genetic mechanism associated with the increased expression of antioxidative enzymes is predominant in normal cells, whereas in cancer cells, the antioxidants work as scavengers of free radicals and do not induce the expression of protective proteins.

It is worth mentioning that the atmospheric pressure during the flight decreased to 1 hPa. However, it is not possible to draw indisputable conclusions about the influence of pressure on the samples due to the fact that, in our research, the cells were surrounded by the layer of the liquid medium, which remained frozen after the temperature drop. Despite its shortcomings, this method still provides exposure for the decreased pressure to a relatively large extent, which probably influenced the cell homeostasis and, hence, the presented data. Notably, among available literature, low-pressure cultivation have been proven to influence homeostasis, morphology, and proliferation of mammalian cells (Watase et al., 1997), which become more rounded and less spindle-shaped, showing numerous blebs around their margins. Mitochondria and nuclei take more round shape due to edema and cell membrane creating bubbles causing cell death (Lyon et al., 1976).

Remarkably, stratospheric balloon flights are accompanied with overloads. The accelerometer in the gondola shows that ascending and descending velocity is not permanent, and as a result, the biological samples were exposed to overloads. Numerous studies have investigated the effect of overloads on cells. For example, when animal cells are cultured under 10 g (10 g means 10 times higher force than Earth gravity), proliferation is increased by 20–30%, glucose consumption is reduced, and cell migration is inhibited by high overloads. At gravitational stress, cell may move to other metabolic pathways (Tschopp and Cogoli, 1983). On the other hand, overloads work as a natural antioxidant and reduce lipid membrane peroxidation (LPO). Induction of the passive mechanisms of biological membrane protection associated with changes in the phase status of the membrane is the most plausible explanation for the phenomenon observed (Markin, 2016). In our experiment, we noted changes in membrane permeability, which could have been affected by overloads. Zhan et al. (1999) revealed a protective effect of green tea polyphenols on 10 g stress in rats (Zhan et al., 1999); our studies highlighted the value of natural antioxidants as protective agents in the gravitational stress. Furthermore, altered gravity deeply influences numerous biological processes in living organisms and even affects gene expression providing adaptive responses (Genchi et al., 2018). Changes in gravitational values affect cell survival, development, and spatial organization. In addition, the indirect effects of altered gravity, such as those associated with hydrostatic pressure and fluid shear, strongly affect both *in vitro* and *in vivo* systems (Genchi et al., 2016). Hence, to understand these phenomena, further research is necessary.

## Conclusions

In our study, we analyzed changes in the functions of normal and cancer cells that occurred due to exposure to high radiation, overloads, as well as low temperature and pressure during stratospheric flight. Our work has led us to a conclusion that the application of the carefully selected compounds enables us to manipulate cellular stress response depending on the type of cells. Final conclusions about the highest protective potential should be drawn based on the genotoxicity assays and cell death assays. Altogether, these findings suggest that honokiol and catechin have the best protective effect on the normal cells, whereas curcumin and cinnamon act as radio- and light sensitizers increasing the percentage of apoptotic cancer cells and DNA damage. The results constitute a significant step towards the investigation of possible strategies for the cell protection in space environment and provide new insights into the application of the examined compounds for the prevention and treatment of cancer. We believe that our research will remain valuable for resolving the difficulty of the human and biological material protection in space. Owing to its relatively low costs, our approach remains the economic alternative for simulated subcosmic conditions conducted in the laboratory, which require far more expensive, specialized measurements.

## Data Availability

The datasets generated for this study are available on request to the corresponding author.

## Author Contributions

DP was responsible for project administration. DP, AG, PR, and WB wrote the original draft and performed most of investigations. OM helped with the formal analysis. JR performed flow cytometry. AS was responsible for CLSM. MD-Z and PK helped with comet assay. JG was responsible for stratospheric conditions measurements. JK was the supervisor of the experiment and the main reviewer. All authors reviewed the manuscript.

## Funding

The work was created as part of the activity of the Student Research Group “Biology of Cancer Cell” at the Wroclaw Medical University (SKN No. K 148). The publication was prepared under the project financed from the funds granted by the Ministry of Science and Higher Education in the “Regional Initiative of Excellence” programme for the years 2019–2022, project number 016/RID/2018/19, the amount of funding 11 998 121.30 PLN. The research was supported partially by the Statutory Funds of Wroclaw Medical University (Department of Molecular and Cellular Biolgy), funds of Wroclaw University of Science and Technology and by “Najlepsi z Najlepszych 3.0” program No. POWER.Z600.18.002 funded by Polish Ministry of Science and Higher Education. PK is the beneficiary of L’Oreal Poland and the Polish Ministry of Science and Higher Education and START Foundation for Polish Science scholarships. The funder was not involved in the study design, collection, analysis, interpretation of data, the writing of this article or the decision to submit it for publication.

## Conflict of Interest Statement

The authors declare that the research was conducted in the absence of any commercial or financial relationships that could be construed as a potential conflict of interest.
